# Modeling Transient Abnormal Myelopoiesis Using Induced Pluripotent Stem Cells and CRISPR/Cas9 Technology

**DOI:** 10.1016/j.omtm.2020.09.007

**Published:** 2020-09-16

**Authors:** Sonali P. Barwe, Ishnoor Sidhu, E. Anders Kolb, Anilkumar Gopalakrishnapillai

**Affiliations:** 1 Nemours Center for Childhood Cancer Research, A.I. DuPont Hospital for Children, Wilmington, DE 19803, USA; 2 University of Delaware, Newark, DE 19711, USA

**Keywords:** Down syndrome, iPSC, CRISPR/Cas9, leukemia, GATA1s

## Abstract

Approximately 1%–2% of children with Down syndrome (DS) develop acute myeloid leukemia (AML) prior to age 5 years. AML in DS children (ML-DS) is characterized by the pathognomonic mutation in the gene encoding the essential hematopoietic transcription factor *GATA1*, resulting in N-terminally truncated short form of GATA1 (GATA1s). Trisomy 21 and GATA1s together are sufficient to induce transient abnormal myelopoiesis (TAM) exhibiting pre-leukemic characteristics. Approximately 30% of these cases progress into ML-DS by acquisition of additional somatic mutations. We employed disease modeling *in vitro* by the use of customizable induced pluripotent stem cells (iPSCs) to generate a TAM model. Isogenic iPSC lines derived from the fibroblasts of DS individuals with trisomy 21 and with disomy 21 were used. The CRISPR (Clustered Regularly Interspaced Short Palindromic Repeats)/Cas9 system was used to introduce *GATA1* mutation in disomic and trisomic iPSC lines. The hematopoietic stem and progenitor cells (HSPCs) derived from *GATA1* mutant iPSC lines expressed GATA1s. The expression of GATA1s concomitant with loss of full-length GATA1 reduced the erythroid population, whereas it augmented megakaryoid and myeloid populations, characteristic of TAM. In conclusion, we have developed a model system representing TAM, which can be used for modeling ML-DS by stepwise introduction of additional mutations.

## Graphical Abstract

**Figure:**
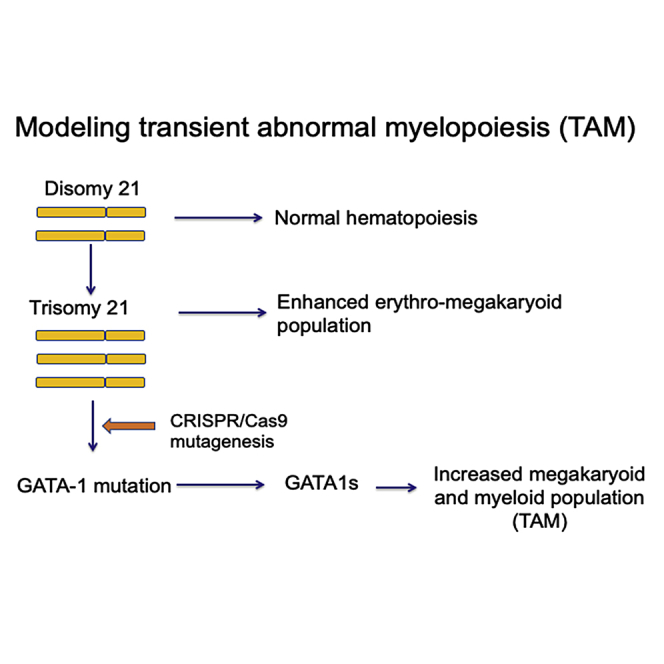

## Teaser

Acute myeloid leukemia in Down syndrome children is characterized by the mutation in *GATA1*, resulting in N-terminally truncated mutant GATA1 (GATA1s). Trisomy 21 and GATA1s together induce transient abnormal myelopoiesis (TAM) exhibiting pre-leukemic characteristics. The expression of GATA1s reduced the erythroid population and augmented megakaryoid and myeloid populations, characteristic of TAM.

## Introduction

Down syndrome (DS) is the most common genetic disorder in humans and is characterized by trisomy of chromosome 21. It is recognized as one of the most prevalent leukemia-predisposing syndromes.^1^ Young children with DS have a 500-fold increased incidence of acute myeloid leukemia (AML),^2^ probably because of the imbalance in the expression of genes, such as *RUNX1*, *DYRK1A* on chromosome 21, which can affect hematopoiesis.^3^^,^^4^ A 4-Mb region on chromosome 21 containing transcription factors *RUNX1*, *ETS2*, and *ERG* was shown to be sufficient for transient abnormal myelopoiesis (TAM) in the presence of a *GATA1* mutation.^5^ Specifically, 1%–2% of DS children develop AML prior to age 5 years.^6^ AML in DS children (ML-DS) is characterized by the pathognomonic mutation in the *GATA1* gene.^6^^,^^7^ In about 1 of 10 DS infants, trisomy 21 and *GATA1* mutation together induce TAM, characterized by an abnormally high population of myeloblasts in the peripheral blood.^8^ Although TAM in most infants is resolved without intervention, approximately 30% of these cases exhibiting preleukemic characteristics progress into ML-DS.

**Figure 1:**
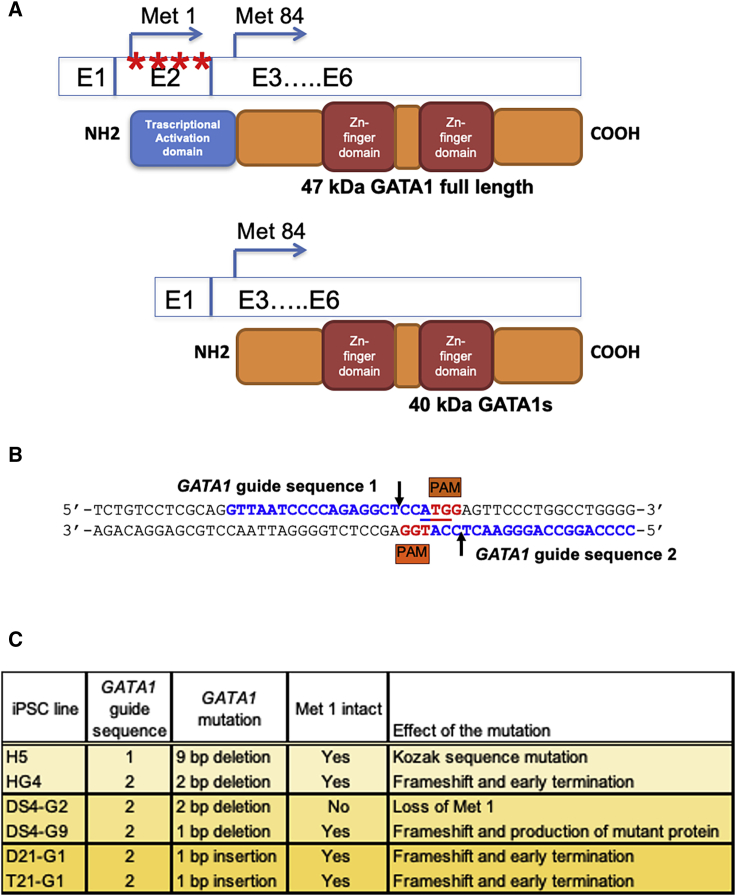
Domain Organization of GATA1 and Nature of GATA1 Mutations in Mutant iPSCs (A) Diagrammatic representation showing *GATA1* mRNA and generation of full-length and mutant GATA1s protein due to the presence of mutations within exon 2 (E2) indicated by red asterisks. Blue box represents N-terminal transcription activation domain that is lost in GATA1s. Red boxes indicate zinc-finger domains. (B) *GATA1* gene sequence showing the two distinct guide sequences used for CRISPR/Cas9 targeting. (C) Description of *GATA1* mutation in different iPSC lines and its effect on protein expression.

*GATA1* gene on the X chromosome encodes the essential hematopoietic transcription factor playing a major role in erythrocyte and megakaryocytic differentiation. Mutations in *GATA1* have been detected in the cells of most TAM and ML-DS patients. However, these mutations are conspicuously absent in other types of leukemia. The majority of the reported mutations (deletion, insertion, missense, or nonsense) in the *GATA1* gene are concentrated in exon 2 coding for the initial 83 amino acids containing the transcriptional activation domain. Exon 1 is non-coding and generates the 5′ untranslated region. The mutations result in the production of N-terminally truncated short form of GATA1 (GATA1s, where s stands for short) protein devoid of exon 2.^9^, ^10^, ^11^ A diagrammatic representation of the modular domains of full-length GATA1 and GATA1s is shown (Figure 1A). Although the DNA binding zinc-finger domains are intact in GATA1s, this truncated protein is deficient in the suppression of E2F target genes such as *MYC* because of loss of protein-protein interaction with E2F^12^ and reduced promoter occupancy in the *MYC* gene.^13^

We employed disease modeling *in vitro* by customizing induced pluripotent stem cells (iPSCs)^14^^,^^15^ by precise gene editing to identify individual and synergistic contribution of trisomy 21 and GATA1s in inducing transient leukemia. Isogenic disomic and trisomic iPSC lines and independent heterogeneous trisomic lines possessing *GATA1* mutation were generated by CRISPR (Clustered Regularly Interspaced Short Palindromic Repeats)/Cas9 methodology. Differentiation of these iPSCs into hematopoietic stem and progenitor cells (HSPCs) showed that GATA1s and trisomy 21 increased the abundance of the megakaryoid and myeloid population, characteristic of TAM. Thus, using CRISPR/Cas9-mediated customized iPSCs, we developed a system to model the stepwise mutagenesis in ML-DS induction.

## Results

### Generation of *GATA1* Mutant Disomic and Trisomic iPSCs by CRISPR/Cas9 Mutagenesis

In order to model the multi-step ML-DS leukemogenesis, we used iPSCs bearing trisomy 21 derived from DS patient fibroblasts (H, DS4, and T21). iPSC line with disomy 21 and isogenic to T21 (D21) was also used to determine the contribution of an extra copy of chromosome 21 in leukemogenesis. The second step in ML-DS leukemogenesis was achieved by *GATA1* mutagenesis using the CRISPR/Cas9 system. *GATA1* mutations in the majority of ML-DS patients cause disruption of initiation codon (Met 1) or introduction of a premature termination codon owing to a frameshift mutation downstream of Met 1 (Figure 1A). Therefore, CRISPR guide sequences that target Met 1, and thereby force translation from an alternate initiation codon (Met 84), were designed to generate the GATA1s protein.

CRISPR guide sequence immediately upstream of Met 1 (referred to as *GATA1* guide sequence 1; Figure 1B), which we employed previously for reassignment of *GATA1* initiation codon in K562 cells,^16^ was used. We initially used iPSC line H derived from the fibroblasts of a DS male individual with a 47, XY+21 karyotype, because *GATA1* is located on the X chromosome and there is a single *GATA1* allele in these cells. Sequence and tracking of indels by decomposition (TIDE) analysis of the CRISPR/Cas9 target region identified clones with either 1-bp insertion or 6-bp deletion upstream of the cut site with efficiency greater than 95%. Both of these mutations did not affect translation from Met 1 and produced only full-length GATA1 protein (data not shown). Interestingly, one clone with a 9-bp deletion resulting in a G-to-A conversion at the highly conserved −6 position within the Kozak consensus sequence was obtained (Clone H5; Figure 1C; Figures S1A and S1B).

The identification of clones with resection upstream of the CRISPR/Cas9 complex cut site such that Met 1 and downstream codons were intact prompted us to design another CRISPR guide sequence downstream of ATG and located on the reverse strand (*GATA1* guide sequence 2; Figure 1B). Using this guide sequence, we identified several clones with Cas9 activity within the coding region. A clone with a 3-bp deletion (c.5-7 “AGT”) accompanied by insertion of “A” at the cut site, resulting in a net 2-bp deletion downstream of ATG, was obtained (Clone HG4; Figure 1C; Figure S1C). A similar mutation with c.5-7 “AGT” replaced by “C” has been reported earlier in a ML-DS patient.^17^ This mutation resulted in a reading frameshift and the introduction of a premature termination codon beyond 37 amino acids.

Because *GATA1* guide sequence 2 yielded higher mutagenesis activity, it was used for *GATA1* mutagenesis in another trisomic iPSC line (DS4), also derived from a male DS individual, similar to iPSC line H. Two DS4 clones with hemizygous loss of Met 1 initiation codon were obtained. These clones had “ATG” disruption and loss of the first initiation codon because of 2-bp deletion downstream of the cut site (Clone DS4-G2; Figure 1C; Figure S2A). Other clones with 2-bp deletion and insertion of “A” upstream of the cut site resulting in net loss of 1 bp were obtained (Clone DS4-G9; Figure 1C; Figure S2B). In spite of the frameshift, no premature termination codon was generated, leading to the production of a mutant protein.

In order to compare the effect of GATA1s on genetically identical backgrounds except for the presence of a third copy of chromosome 21, we utilized the isogenic disomic/trisomic pair of iPSCs. D21 and T21 iPSC lines were derived from a female DS individual and possess two *GATA1* alleles. The majority of the mutant clones produced monoallelic 1-bp insertion downstream of ATG, resulting in reading frameshift and appearance of a premature termination codon after 38^th^ amino acid (Clones T21-G1 and D21-G1; Figure 1C; Figures S3A and S3B, respectively).

### Detection of GATA1s Protein in HSPC Lysate

**Figure 2:**
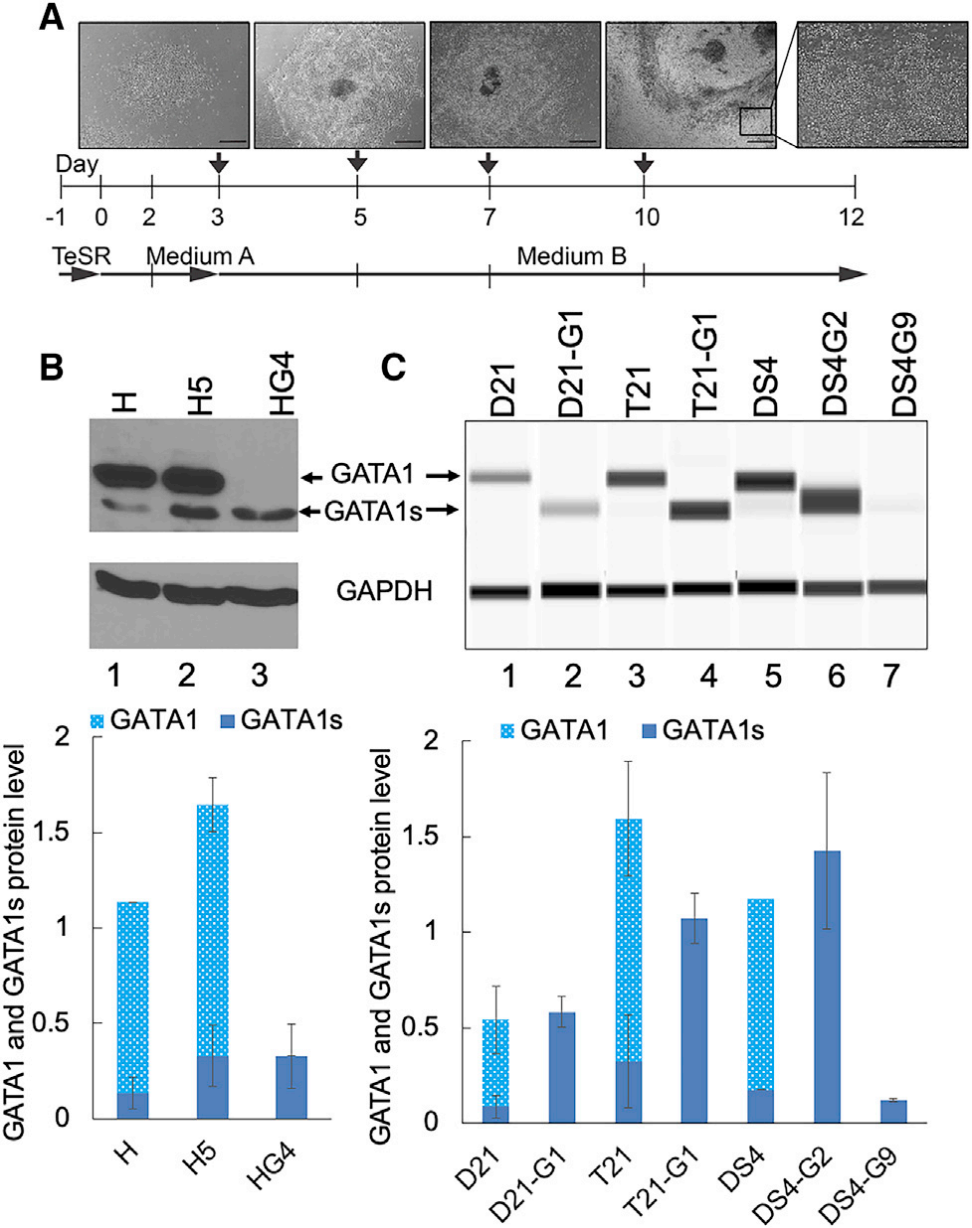
Hematopoietic Differentiation of Disomic and Trisomic iPSCs with WT and Mutated GATA1 and the Expression Level of GATA1 and GATA1s Protein (A) Hematopoietic differentiation schema with images showing the colony morphology at times indicated by vertical arrows. Half media changes are indicated by vertical lines. Scale bars: 500 μm. (B) Representative western blots showing the level of full-length and short form of GATA1 in HSPCs. GAPDH was used as a loading control. The graph shows the relative levels of GATA1 and GATA1s normalized to GAPDH from three independent experiments. (C) Immunoblots using the automated western blotting system Wes in band view to show the difference in the mobility of GATA1 and GATA1s. The graph shows the relative levels of GATA1 and GATA1s protein normalized to GAPDH and total protein from three independent experiments. In (B) and (C), error bars denote SE of the mean.

GATA1 protein is not expressed in iPSCs (data not shown). To determine the expression of GATA1 or GATA1s in mutant clones with frameshift mutation or initiation codon deletion, we differentiated these mutant iPSC lines into HSPCs (Figure 2A). HSPCs collected at 10 days post differentiation were lysed and subjected to immunoblot analysis. The HSPCs derived from disomic and trisomic iPSCs with wild-type (WT) *GATA1* showed expression of full-length GATA1 protein and a small amount of GATA1s (Figures 2B, lane 1, and 2C, lanes 1, 3, and 5). Trisomic HSPCs with WT *GATA1* expressed a higher level of GATA1 and GATA1s protein compared with disomic HSPCs (Figure 2C, lanes 1 and 3).

*GATA1* mutant HSPCs with either frameshift or initiation codon deletion lacked the expression of full-length GATA1 (Figures 2B, lane 3, and 2C, lanes 2, 4, 7, and 8). These HSPCs exclusively expressed GATA1s, albeit at variable levels. Interestingly, the Kozak mutant H5 HSPCs expressed full-length GATA1 but also showed GATA1s levels comparable with the frameshift mutant HG4 (Figure 2B, compare lanes 2 and 3). Isogenic trisomic HSPCs with mutant *GATA1* expressed more GATA1s compared with disomic HSPCs possessing *GATA1* mutation (Figure 2C, lanes 2 and 4), consistent with a previous report that trisomy 21 upregulates the expression of GATA1s.^5^

Although D21-G1 and T21-G1 iPSCs had heterozygous *GATA1* mutation, HSPCs derived from both of these lines showed no full-length GATA1 protein, likely because of the inactivation of the X chromosome bearing WT *GATA1*. DS4-G2 HSPCs expressed 12-fold higher GATA1s than DS4-G9 HSPCs (Figure 2C, compare lanes 6 and 7), probably because of the distinct mutations in these clones. Although DS4-G2 had deletion of Met 1, DS4-G9 had a frameshift mutation resulting in the production of a mutant protein.

### Hematopoietic Differentiation of iPSC Lines with or without Trisomy 21 and/or *GATA1* Mutation

To determine the role of trisomy 21 and *GATA1* mutation on the stages of hematopoietic development, we analyzed iPSCs cultured in differentiation media for the early mesoderm markers HAND1 and Brachyury on day 3 (Figure S4). D21, T21, and T21-G1 iPSCs showed staining for either of these markers, indicating that either trisomy 21 or *GATA1* mutation did not affect early mesoderm differentiation. We also analyzed the expression of hemangioblast markers CD31 and podocalyxin (Podxl) by flow cytometry at different time points during differentiation (Figure S5). There were no significant differences between the percentages of CD31^+^ and Podxl^+^ cells between D21, D21-G1, T21, and T21-G1 at early time points, indicating that early hematopoietic development was not affected by either trisomy 21 or mutated *GATA1*. Similarly, the evaluation of cell percentages positive for hematopoietic cell lineage markers CD90, CD34, CD41, CD43, and CD45 did not show significant differences between disomic and trisomic lines with or without *GATA1* mutation (Figure S6). Taken together, these results are in agreement with previous studies showing no alterations in the generation of hematopoietic progenitors by trisomy 21^18^ or by GATA1s.^19^

We then tested the hematopoietic colony-forming potential of HSPCs derived from the isogenic disomic and trisomic iPSC lines with WT or mutated *GATA1*. HSPCs with WT *GATA1* produced three types of colonies: BFU-E (burst-forming unit-erythroid), CFU-GM (colony-forming unit-granulocyte, macrophage), and CFU-GEMM (colony-forming unit-granulocyte, erythrocyte, macrophage, megakaryocyte) (Figure S7). The trisomic HSPCs with WT *GATA1* generated more erythroid, myeloid, and mixed colonies compared with disomic HSPCs with WT *GATA1*, possibly because of the stimulation in the number of hematopoietic progenitor cells by trisomy 21 as shown previously.^5^^,^^19^^,^^20^ The presence of *GATA1* mutation, irrespective of ploidy, hampered the generation of erythroid or mixed colonies, whereas the number of myeloid colonies produced was greatly increased, consistent with a prior report.^19^

### Analysis of Erythroid, Myeloid, and Megakaryoid Populations in Disomic and Trisomic iPSCs with or without *GATA1* Mutation

**Figure 3:**
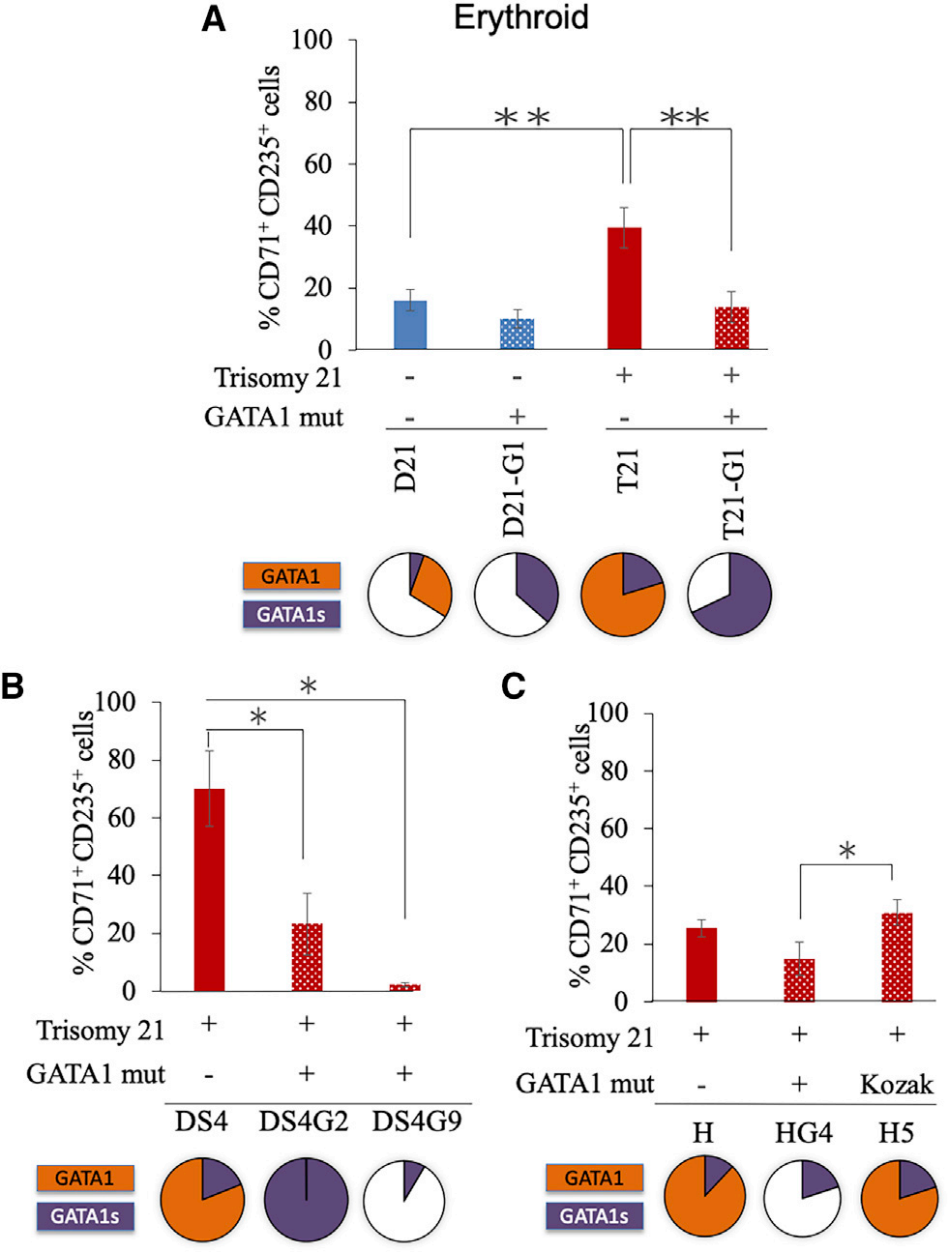
Effect of *GATA1* Mutation on Erythroid Population Graphs show the percentage of erythroid population in HSPCs generated from (A) isogenic disomic and trisomic iPSC lines with or without *GATA1* mutation, (B) trisomic iPSC line DS4 with or without GATA1 mutation, (C) trisomic iPSC line H with or without GATA1 mutation. Average data from 3–12 independent experiments are plotted. Error bars indicate SE of the mean. ∗p < 0.05, ∗∗p < 0.005. The pie charts shown below the graphs represent the comparative levels of GATA1 (orange) and GATA1s (purple) proteins.

Following hematopoietic differentiation of the iPSC line panel, the percentage of the erythroid population characterized as CD71^+^ CD235^+^ was determined by multi-dimensional flow cytometry (Figure S8A). A significant increase in the percentage of erythroid population was observed in trisomy 21 HSPCs with full-length GATA1 compared with the isogenic disomy 21 HSPCs (Figure 3A, compare bars 1 and 3; p < 0.005), confirming that the extra copy of chromosome 21 stimulates erythroid expansion as reported previously.^5^^,^^18^^,^^19^^,^^21^^,^^22^ The introduction of the *GATA1* mutation resulted in a reduction in the erythroid population in both disomic (Figure 3A, compare bars 1 and 2) and trisomic HSPCs (Figures 3A, compare bars 3 and 4, 3B, compare bars 1 and 2–3, and 3C, compare bars 1 and 2), consistent with a prominent role of *GATA1* in erythroid development. The Kozak sequence mutant of *GATA1* did not affect the erythroid development (Figure 3C, compare bars 1 and 3).

**Figure 4:**
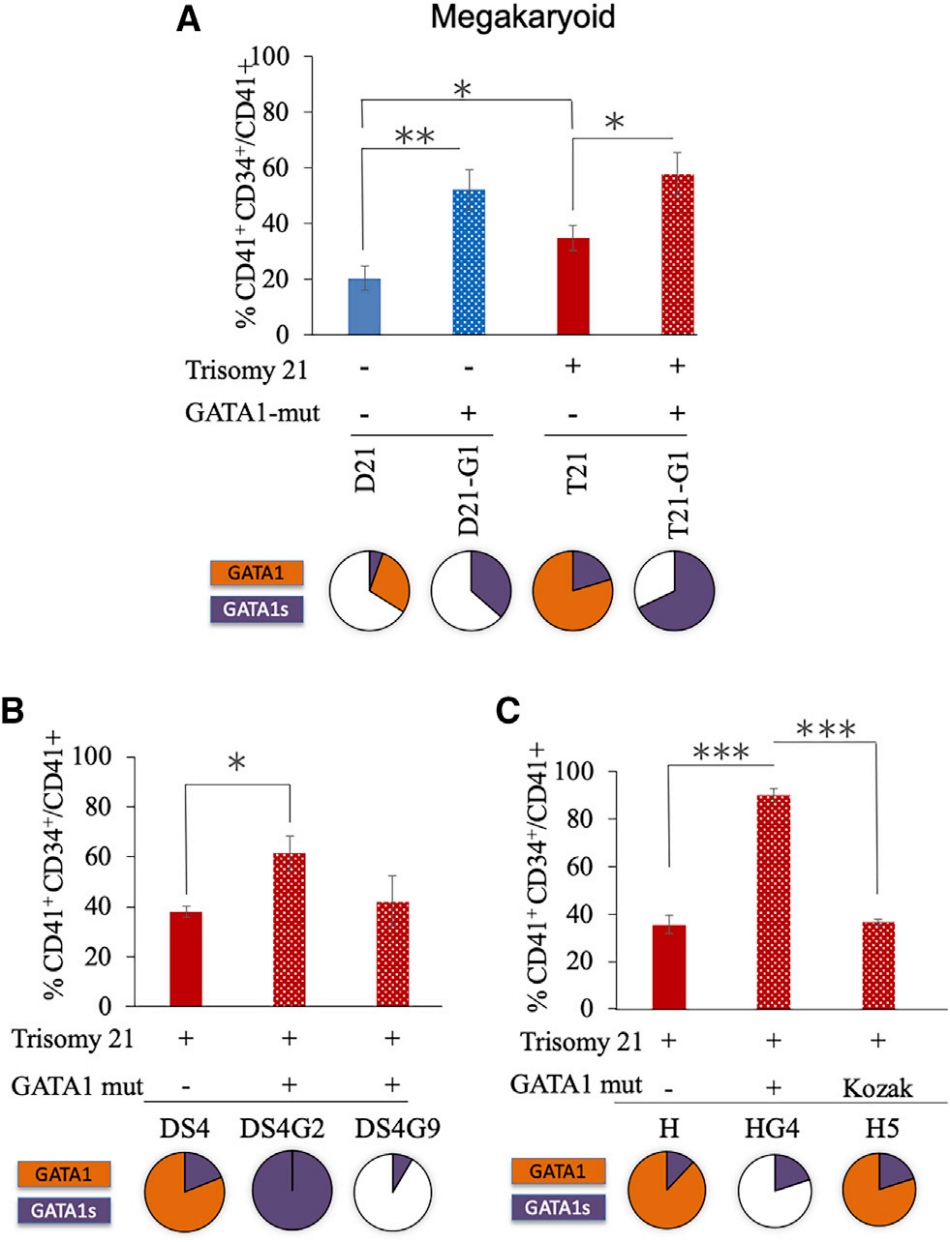
Effect of *GATA1* Mutation on Megakaryoid Population Graphs show the percentage of megakaryoid population (calculated as CD34^+^CD41^+^/CD41^+^ × 100) in HSPCs generated from (A) isogenic disomic and trisomic iPSC lines with or without *GATA1* mutation , (B) trisomic iPSC line DS4 with or wihtout GATA1 mutation, (C) trisomic iPSC line H with or without GATA1 mutation. Average data from 3–12 independent experiments is plotted. Error bars indicate SE of the mean. ∗p < 0.05, ∗∗p < 0.005, ∗∗∗p < 0.0005. The pie charts shown below the graphs represent the comparative levels of GATA1 (orange) and GATA1s (purple) proteins.

A percentage of the megakaryoid population was analyzed by flow cytometry using CD34 and CD41 markers. During megakaryocytic differentiation, the progenitor cells co-express CD34 and CD41 transiently before producing CD34^−^CD41^+^ mature megakaryocytic cells.^23^^,^^24^ Therefore, the percentage of immature megakaryoblasts was calculated as the ratio of the CD34^+^CD41^+^ population within the total CD41^+^ population as described previously^5^ (Figure S8B). The *GATA1* mutated HSPCs showed enhanced percentage of immature megakaryoblasts, which is the hallmark of transient leukemia in DS children. This pattern was not only observed in the three trisomic iPSC clones carrying the *GATA1* mutation (Figures 4A, compare bars 3 and 4, 4B, compare bars 1 and 2–3, and 4C, compare bars 1 and 2) but also was seen in a disomic iPSC line with *GATA1* mutation (Figure 4A, compare bars 1 and 2). An increased percentage of immature megakaryoblasts was observed when comparing disomic versus trisomic HSPCs without *GATA1* mutation (Figure 4A, compare bars 1 and 3), consistent with a previous report that trisomy 21 stimulates erythro-megakaryocytic expansion.^21^ Clone H5 was significantly different from clone HG4 with respect to the megakaryoid population (Figure 4C, compare bars 2 and 3) suggesting that, unlike frameshift mutant *GATA1*, the Kozak mutant of *GATA1* failed to alter the megakaryoid population.

**Figure 5:**
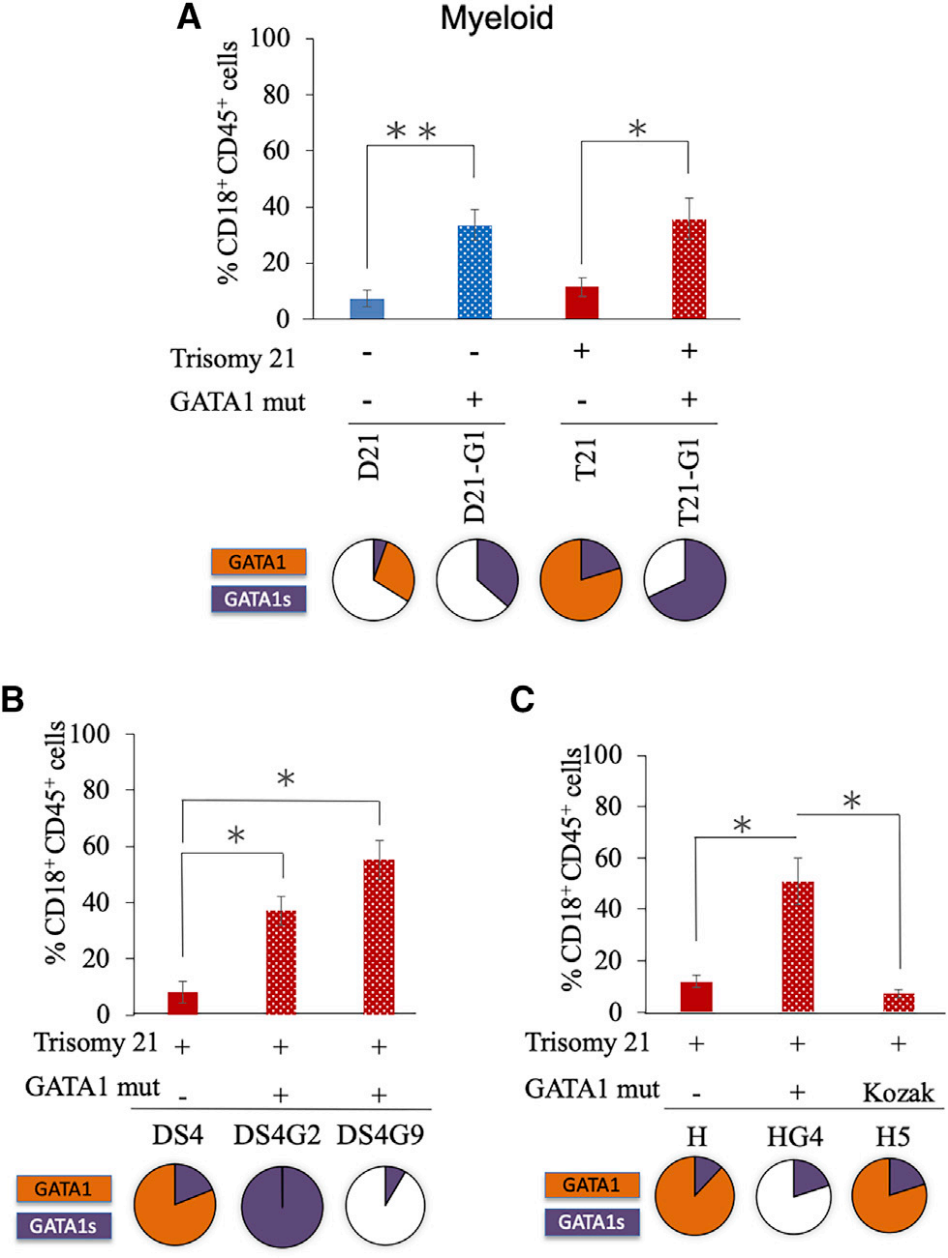
Effect of *GATA1* Mutation on Myeloid Population Graphs show the percentage of myeloid population in HSPCs generated from (A) isogenic disomic and trisomic iPSC lines with or without *GATA1* mutation , (B) trisomic iPSC line DS4 with or without GATA1 mutation, (C) trisomic iPSC line H with or without GATA1 mutation. Average data from 3–12 independent experiments are plotted. Error bars indicate SE of the mean. ∗p < 0.05, ∗∗p < 0.005. The pie charts shown below the graphs represent the comparative levels of GATA1 (orange) and GATA1s (purple) proteins.

To determine the percentage of the myeloid population, we stained cells with CD18 and CD45 as shown in representative plots (Figure S8C). Unlike the effect of trisomy 21 on erythro-megakaryocytic development, trisomy 21 did not have a significant effect on the growth of the myeloid population (Figure 5A, compare bars 1 and 3). Similar to the effect on the megakaryoid population, *GATA1* mutated HSPs showed a higher percentage of myeloid cell population in all three sets of iPSC lines (Figure 5A, compare bars 3 and 4, 5B, compare bar 1 with bars 2–3, and 5C, compare bars 1 and 2). This increased preponderance of the myeloid population was also seen in the *GATA1* mutated iPSC line with disomy 21 (Figure 5A, compare bars 1 and 2). *GATA1* Kozak mutated HSPCs did not show significant alterations in the myeloid population compared with HSPCs with WT *GATA1* (Figure 5C, compare bars 1 and 3).

We also cultured the HSPCs in lineage-specific media and stained the erythroid, megakaryocytic, and myeloid cell populations with May-Grünwald-Giemsa stain. Erythroid differentiation was severely hampered in *GATA1* mutated HSPCs, whereas no significant differences in the morphology of megakaryocytic and myeloid populations were observed (Figure S9), coherent with previous studies.^24^^,^^25^

## Discussion

Children with DS are uniquely predisposed to AML, although the relative risk for solid tumors is lower than in the general population. The ML-DS patients also possess the pathognomonic mutation in *GATA1*; such mutations are absent in other subtypes of myeloid leukemia.^25^ Therefore, attempts have been made to understand the synergy between the unique combination of trisomy 21 and *GATA1* mutations in inducing leukemia. In this study, using HSPCs derived from isogenic disomic and trisomic iPSCs bearing WT or CRISPR/Cas9-induced mutated *GATA1*, we show that the extra copy of chromosome 21 altered erythroid differentiation, and the *GATA1* mutation resulted in reduced percentage of the erythroid population, while enhancing megakaryoid and myeloid populations. Our results are consistent with those obtained by using non-isogenic patient-derived iPSCs and other methods of genome editing, such as zinc-finger nucleases or TALENs,^5^^,^^19^^,^^22^ highlighting the utility of this approach for the stepwise modeling of multi-factorial diseases.

The region upstream of Met 1 in the *GATA1* gene matches the conserved Kozak sequence at −6, −4, −2, −1, and +4 positions, whereas Met 84 matches only at −4 and +4 sites. We postulated that the preferential translation from Met 1 site could be because of this difference in the Kozak consensus loci,^16^ and that disruption of the Kozak site preceding Met 1 may hinder transcription from this start site. Mutations in the highly conserved positions within the Kozak sequence, specifically G-to-C conversion at position −6 relative to “ATG,” have been shown to alter gene translation, leading to disease manifestation.^26^ Such a *GATA4* mutation reduced GATA4 protein level, resulting in atrial septal defect,^27^ and led to β-thalassemia when present in the *β-globin* gene.^28^ Trisomic clone H5, which carried a G-to-A conversion at the highly conserved −6 position, showed an increase in the GATA1s protein (Figure 2B, compare lanes 1 and 2), while the full-length GATA1 protein level was not affected, indicating that there was a small shift toward translation initiation from Met 84 instead of Met 1. No changes in the erythroid, megakaryoid, and myeloid populations were observed following hematopoietic differentiation of this clone, indicating that the increase in GATA1s protein in the presence of full-length GATA1 was not sufficient to trigger alterations indicative of TAM.

Previous reports indicated that the altered dosage of genes on the extra chromosome 21 elevates the level of GATA1s protein.^5^^,^^29^ Consistent with these reports, we observed increased GATA1s protein in T21-G1 compared with D21-G1 HSPCs. The nature of the *GATA1* mutation also dictated levels of GATA1s. *GATA1* mutant clone with ablation of the Met 1 (DS4-G2) produced more GATA1s protein compared with clones with insertion or deletion in exon 2, resulting in frameshift and a premature termination codon prior to Met 84. *GATA1* frameshift mutation that did not result in a premature termination codon produced the least amount of GATA1s (DS4-G9). iPSC lines with mutated *GATA1* (except the Kozak mutation) had reduced erythroid population and increased megakaryoid and myeloid populations irrespective of the level of GATA1s protein. It was shown earlier that the level of GATA1s protein in TAM blasts is variable and negatively correlates with progression to AML and poor prognosis.^30^ Whether the HSPCs derived from these clones exhibit differential disease severity remains to be determined.

GATA1 is a transcription factor with demonstrated function in erythrocyte differentiation and other hematopoietic lineages, including megakaryocytes.^31^ Expression of N-terminally truncated GATA1 in mice induced abnormal accumulation of megakaryocytic progenitors in the absence of chromosomal aneuploidy.^32^ The expression of GATA1s reduced erythroid lineage cells, whereas it augmented megakaryoid and myeloid lineages in both disomy 21 and trisomy 21 backgrounds. Kadri et al.^33^ showed that the LXCXE motif within the GATA1 N terminus (81–85 amino acids) was important for binding of pRbE2F to GATA1, which is necessary for the maturation of erythrocytes. Subsequent studies have also highlighted the importance of the GATA1 N terminus in erythrocyte development.^19^^,^^34^ Lack of LXCXE motif in GATA1s is shown to prevent a direct interaction of GATA1s to E2F, resulting in hyperproliferation of megakaryocytes.^12^ However, the exact mechanism by which GATA1s induces abnormal megakaryopoiesis remains elusive.

These two mutagenic events, trisomy 21 and GATA1s, are not sufficient for ML-DS leukemogenesis. Whole-genome and whole-exome sequencing studies identified recurrent somatic mutations in ML-DS, which were not detected in TAM.^35^, ^36^, ^37^ The prevalence of these putative driver mutations in ML-DS was the basis of the general agreement in the field that acquisition of additional somatic mutations drives TAM to transform into ML-DS. Our long-term goal is to use these human isogenic disomic and trisomic iPSC lines for sequential introduction of ML-DS-specific somatic mutations to model ML-DS and determine the individual and synergistic effects of trisomy 21, GATA1s, and additional somatic mutations in the induction of megakaryoid and myeloid hyperproliferation.

## Materials and Methods

### iPSC Lines and Culture

Isogenic iPSC lines derived from the fibroblasts of a DS patient with trisomy 21 (T21C1; referred to as T21 in this study) and with disomy 21 (T21C5; referred to as D21 in this study) in which chromosome 21 was spontaneously lost during passaging (described in Chen et al.^38^) were obtained from RUCDR Infinite Biologics at Rutgers University, a part of NIH Center for Regenerative Medicine. The DS4 trisomic iPSC line was obtained from WiCell (Madison, WI, USA). DS2-iPS10, an iPSC line derived from DS patient fibroblast (referred to as H in this study), was a kind gift from Prof. George Daley, Children’s Hospital, Harvard University (Boston, MA, USA).^39^

Routine iPSC culture was done in complete mTeSR1 media (Stem Cell Technologies, Ontario, CA, USA) on plates coated with Matrigel (Corning, Tewksbury, MA, USA). Subculturing was performed every 4–6 days by dissociating iPSC colonies by incubation with cell dissociation agent (STEMCELL Technologies) for 3 min followed by scraping the colonies into the mTeSR1 media. Whenever colonies were revived from liquid nitrogen, mTeSR1 was supplemented with 10 μM Rho Kinase inhibitor, Y27632 (Cayman, Ann Arbor, MI, USA) overnight before continuing the culture in fresh mTeSR1 media. Cells were tested for their pluripotency by determining the expression of pluripotency markers TRA-1-60 and SSEA4 (BioLegend, San Diego, CA, USA) by flow cytometry using Novocyte 3000 flow cytometer (ACEA Biosciences, San Diego, CA, USA) (data not shown). Bioauthentication was performed to confirm the ploidy^40^ and integrity of the iPSC lines using the AmpFLSTR Identifiler PCR Amplification kit (Thermo Fisher Scientific) (Figure S10).

### CRISPR Design and Cloning

CRISPR guides targeting Met 1 of *GATA1* were designed with the help of an algorithm developed by Ran et al.^41^ *In silico* off-target analysis using CRISPOR (http://crispor.tefor.net/)^42^ showed potential off-targets to be 0 for identical and up to 1-bp mismatch for both guide sequences targeting *GATA1*. Oligonucleotides were obtained from IDT (Coralville, IA, USA) and cloned into pSpCas9(BB)-2A-GFP (PX458, plasmid #48138; Addgene, Watertown, MA, USA), a generous gift from Dr. Feng Zhang,^41^ following standard protocol. Cells for transfection were grown for 48 h. Four hours prior to transfection, fresh mTeSR1 media containing 10 μM Y27632 was added. Transfection was performed using a 4D nucleofector system and P3 Primary Cell 4D-Nucleotransfector X Kit L (Lonza, Basel, Switzerland) with 0.75 × 10^5^ cells and 3.5 μg of plasmid using the program CA189. Transfected cells were cultured in 12-well plates. Two days posttransfection, GFP^+^ cells were sorted into 96-well plates carrying media with SMC4 cocktail containing 5 μM thiazovivin, 1 μM CHIR99021, 0.4 μM PD0325901, 2 μM SB431542 (Cayman), and 50 μg/mL gentamycin (Thermo Fisher, Waltham, MA, USA) using BD FACSAria III (BD Biosciences, Franklin Lakes, NJ, USA). Individual colonies were expanded for freezing and DNA analysis. Genomic DNA was isolated using MicroDNA kit (QIAGEN, Germantown, MD, USA) and used as a template for PCR using primers flanking the guide sequence. PCR products were Sanger sequenced, and the sequence was analyzed using TIDE, a free web-based software tool (https://tide.nki.nl).^43^ Clones showing desired mutation were further expanded and analyzed. *GATA1* mutation was confirmed again via Sanger sequencing.

### Hematopoietic Differentiation

iPSC colonies bearing desired mutation were differentiated using STEMdiff Hematopoietic kit obtained from STEMCELL Technologies (Catalog No. 05310) following the manufacturer’s protocol (outlined in Figure 2A). Thirty to 80 uniform sized colonies were plated in a six-well plate, and the next day media were exchanged with hematopoietic differentiation medium A, and on day 2 half media change was carried out with medium A. On day 3, medium A was removed, medium B added, and half media change was carried out on days 5, 7, and 10.

### Western Blot Analysis

HSPCs collected in the supernatant on day 10 of hematopoietic differentiation were lysed in SDS lysis buffer. Total protein in cell lysates was estimated using Bradford assay (Bio-Rad, Hercules, CA, USA). 25 μg of protein lysate was separated on a 10% SDS-PAGE gel, transferred onto a polyvinylidene fluoride (PVDF) membrane (GE Healthcare, Chicago, IL, USA), blocked with 5% non-fat dry milk, and stained overnight with GATA1 or GAPDH antibody (Cell signaling Technology, Danvers, MA, USA). After horseradish peroxidase (HRP)-conjugated secondary antibody incubation, blots were developed using a chemiluminescent lightning system according to the manufacturer’s recommendations (GE Healthcare). Quantitation was performed using the Bio-Rad GeneTools software.

Alternatively, Wes system (ProteinSimple) was used for automated western blot analysis according to the manufacturer’s instructions using a 12- to 230-kDa Separation Module (ProteinSimple SM-W001) and the anti-Rabbit Detection Module (ProteinSimple DM-001). HSPCs were lysed in Pierce RIPA buffer (Thermo Fisher), sonicated, and cleared by centrifugation at 14,000 × *g* for 15 min. Supernatants were collected, and protein was estimated. 0.125 μg protein was loaded per capillary. Quantitation was performed using Wes. Normalization to total protein using the Total Protein Detection Module in Wes (DM-TP010) was used to confirm the validity of GAPDH as a loading control.

### Flow Cytometry

The erythroid, megakaryoid, and myeloid populations in supernatant HSPCs collected on day 12 of hematopoietic differentiation were characterized by flow cytometry using lineage-specific markers. Cells (50,000) were resuspended in 100 μL phosphate-buffered saline (PBS) containing 1% fetal bovine serum (staining solution) and stained using Pacific blue-conjugated CD235 and phycoerythrin (PE)-conjugated CD71 for analysis of the erythroid population, fluorescein isothiocyanate (FITC)-conjugated CD34 and PE-conjugated CD41 for analysis of the megakaryoid population, or allophycocyanin (APC)-conjugated CD18 and Pacific blue-conjugated CD45 for myeloid population for 15 min in the dark. At the end of the incubation, 900 μL staining solution was added. Cells were centrifuged at 500 × *g* for 5 min and resuspended in 100 μL of staining solution. Samples were acquired on a Novocyte flow cytometer. The forward scatter versus side scatter was used for gating live cells (Figure S11A). This gate captured greater than 96% of viable cells based on staining with Calcein violet 450 AM (Catalog No. 65-0854-39; Thermo Fisher Scientific) (Figure S11B). Therefore, cells in the “live” gate based on forward and side scattering (Figure S11C) were gated for singlet population (Figure S11D). Discriminating quadrant gates were set using respective isotype control antibodies for each fluorophore (Figure S11E).

### Statistical Analysis

For the analysis of erythroid, megakaryoid, and myeloid populations, differentiation experiments were repeated 3–12 times, and statistical significance of the differences in percentages between two iPSC lines was determined by two-tailed Student’s t test with unequal variance.

## Author Contributions

S.P.B., E.A.K., and A.G. conceptualized and designed the work; S.P.B., I.S., and A.G. performed experiments and acquired data; S.P.B., I.S., E.A.K., and A.G. analyzed and interpreted data; S.P.B. and A.G. wrote the manuscript; all authors approved the manuscript.

## Conflicts of Interest

The authors declare no competing interests.

## Supplemental Information

Document S1. Supplemental Materials and Methods and Figures S1–S11

Document S2. Article plus Supplemental Information
