# Supplementary material for: Modeling Transient Abnormal Myelopoiesis Using Induced Pluripotent Stem Cells and CRISPR/Cas9 Technology
Source: Mol Ther Methods Clin Dev. 2020 Sep 16;19:201–9. doi: 10.1016/j.omtm.2020.09.007 (PMC7558799; doi:10.1016/j.omtm.2020.09.007)
Supplement: Document S1. Supplemental Materials and Methods and Figures S1–S11 [file mmc1.pdf]

OMTM, Volume *19*

Supplemental Information

Modeling Transient Abnormal Myelopoiesis Using Induced Pluripotent Stem Cells and CRISPR/Cas9 Technology

Sonali P. Barwe, Ishnoor Sidhu, E. Anders Kolb, and Anilkumar Gopalakrishnapillai

Supplementary Methods

Bioauthentication and ploidy analysis

Bioauthentication using the AmpFLSTR Identifiler PCR Amplification kit (ThermoFisher Scientific) (Supplementary Fig. 1) was performed to confirm the ploidy and integrity of the iPSC lines. Among the 15 polymorphic microsatellite markers included in this panel, the D21S11 locus located on chromosome 21 was used for discrimination of trisomy 21 vs disomy 21 based on the number of peaks and peak height as described previously [1]. The karyotype analysis of the iPSC lines was performed by the laboratories from which the lines were procured. For ploidy characterization of the iPSC lines derived from the parental iPSC lines by CRISPR/Cas9 genome engineering, we utilized the D21S11 probe to differentiate between disomy and trisomy 21 depending on the number of peaks at the locus (2 peaks for disomy, 3 peaks for trisomy) or peak height ratio (1:1 for disomy, 2:1 for trisomy). Using a number of patient samples with disomy or trisomy 21, we trained an investigator for data analysis. Following training, the blinded investigator was able to correctly identify the ploidy of chromosome 21 with 100% accuracy.

Analysis of hemangioblast and hematopoietic markers

Cells at different time points were washed with 1x DPBS and treated with Accutase™ (Stem Cell Technologies) for 5 min at 37°C to dissociate into single cells. Cells were centrifuged at 300g for 5 min and resuspended in 100 µl phosphate buffered saline (PBS) containing 1% FBS. The cells were stained with hemogenic endothelium markers (PB-conjugated CD31 and FITC-conjugated Podxl) and hematopoietic markers

(FITC-conjugated CD90, Brilliant Violet 785-conjugated CD34, Brilliant Violet 605-conjugated CD45, PE-conjugated CD41 and APC-conjugated CD43). All antibodies were obtained from Biolegend.

Colony-forming unit assay

To determine the lineage-potential of hematopoietic cells, 500 hematopoietic stem and progenitor cells collected at day 10 post hematopoietic differentiation were cultured in MethoCult™ SF H4636 (Stem Cell Technologies) for 12 additional days. The different colonies (CFU-GEMM, CFU-GM and BFU-E) were identified and counted using EVOS M5000 imaging system.

May-Grünwald-Giemsa staining

50,000 hematopoietic stem and progenitor cells were resuspended in megakaryoid, myeloid or erythroid lineage expansion media (StemCell Technologies) and continued in culture in 96 well plate. Media was changed every other day and on day 12, 100,000 cells were resuspended in 10 µL of PBS containing 1% FBS. Cells were airdried and stained with May-Grünwald-Giemsa (Sigma) as per manufacturer's instruction. Slides were mounted with 50% glycerol and imaged using EVOS M5000 imaging system.

Immunofluorescence analysis of mesodermal markers

iPSC colonies were plated on Matrigel coated coverslips in mTeSR1 media for 24 hours. Next day media was exchanged with hematopoietic differentiation media A. On

day 3, differentiating colonies were fixed and stained with anti-human Brachyury NL557-conjugated goat IgG and/or anti-human HAND1 NL637-conjugated goat IgG (R&D systems, Minneapolis, MN). The cells were imaged using EVOS M5000 imaging system (Thermo Fisher Scientific).

Supplementary Fig. 1

A

GATA1 guide sequence 1 in H –H5

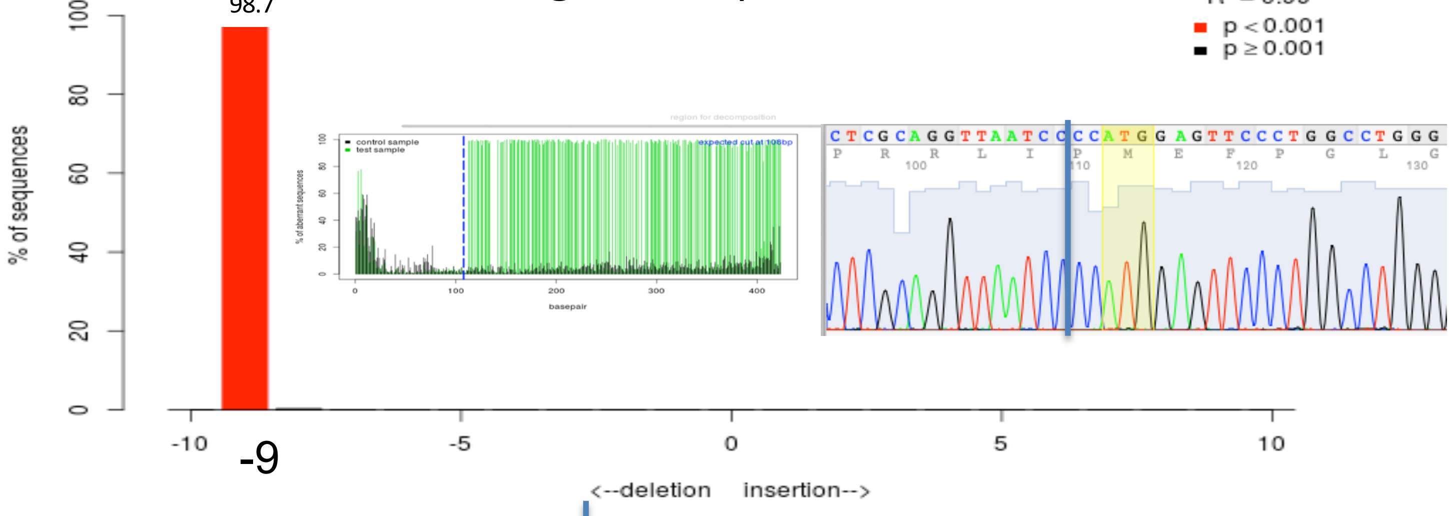

5′ CTCGCAG**GTTAATCCCCAGAGGCTCCATGG**AGTTCCCTGGCCTGGGG -3′ Wild-type
5′ CTCGCAG**GTTAATCC---------CCATGG**AGTTCCCTGGCCTGGGG -3′ Allele 1

B

**GCCACCATGG**	Kozak consensus sequence
**GGCTCCATGG**	Kozak sequence in H (wild-type)
ATCCCC**ATGG**	**Kozak sequence in H5**

C

GATA1 guide sequence 2 in H –HG4

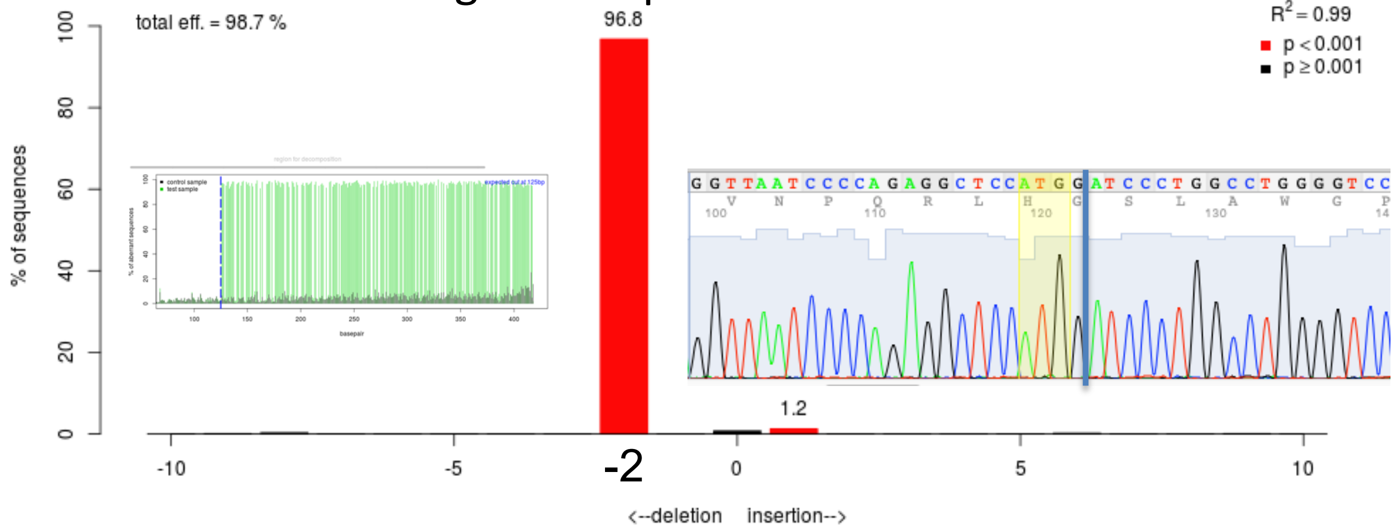

CTCGCAGGTTAATCCCCAGAGGCT**CCATGGAGTTCCCTGGCCTGGGG** Wild-type
CTCGCAGGTTAATCCCCAGAGGCT**CCATGGA--TCCCTGGCCTGGGG** Allele 1

Supplementary Fig. 1 Analysis of *GATA1* mutation in iPSC line H. A) and **C)** Plots of TIDE (Tracking of Indels by Decomposition) analysis showing 98.9% allelic mutation frequency of a clone with 9 bp deletion at chromosomal level and 98.7% mutation frequency in a clone with 2 bp deletion in clone H using guides 1 and 2 respectively. Graph inserts show the presence of sequence aberrations in relation to the CRISPR/Cas9 cut site. Sanger sequencing chromatograms showing the sequence around the cut site (marked with a blue line) are provided. Sequence alignment in the bottom shows the allelic analysis of mutant and wild-type alleles. **B**) Kozak sequences flanking the initiation codon in wild-type and mutated iPSCs and their identity with the Kozak consensus sequence is shown.

Supplementary Fig. 2

A

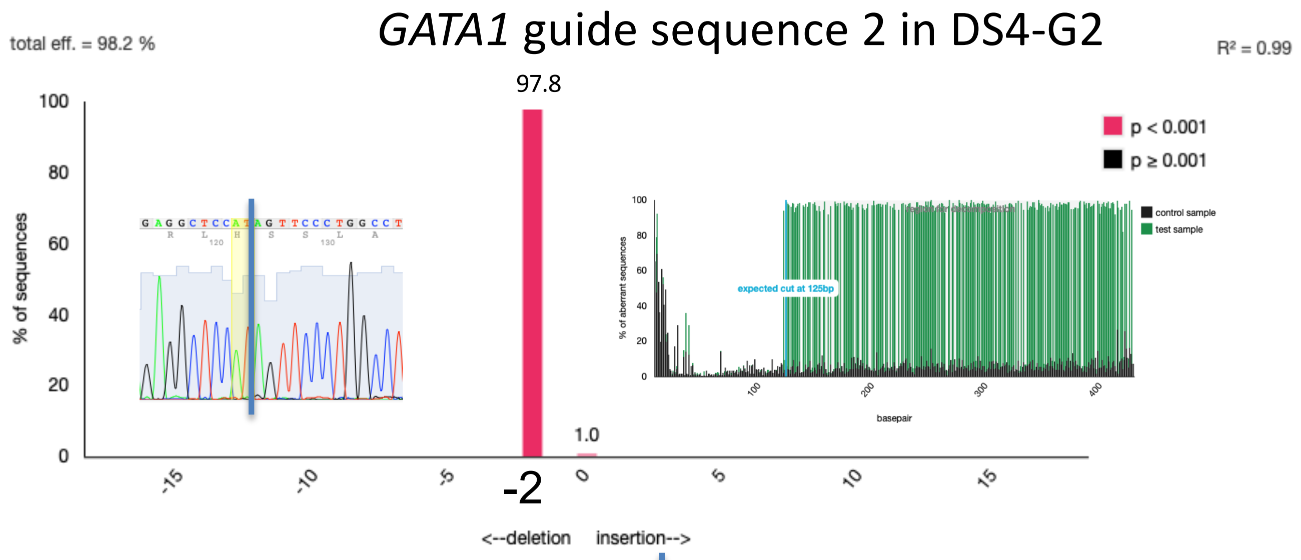


```
CTCGCAGGTTAATCCCCAGAGGCTCCATGGAGTTCCCTGGCCTGGGG Wild-type
CTCGCAGGTTAATCCCCAGAGGCTCCAT--AGTTCCCTGGCCTGGGG Allele 1
```

B

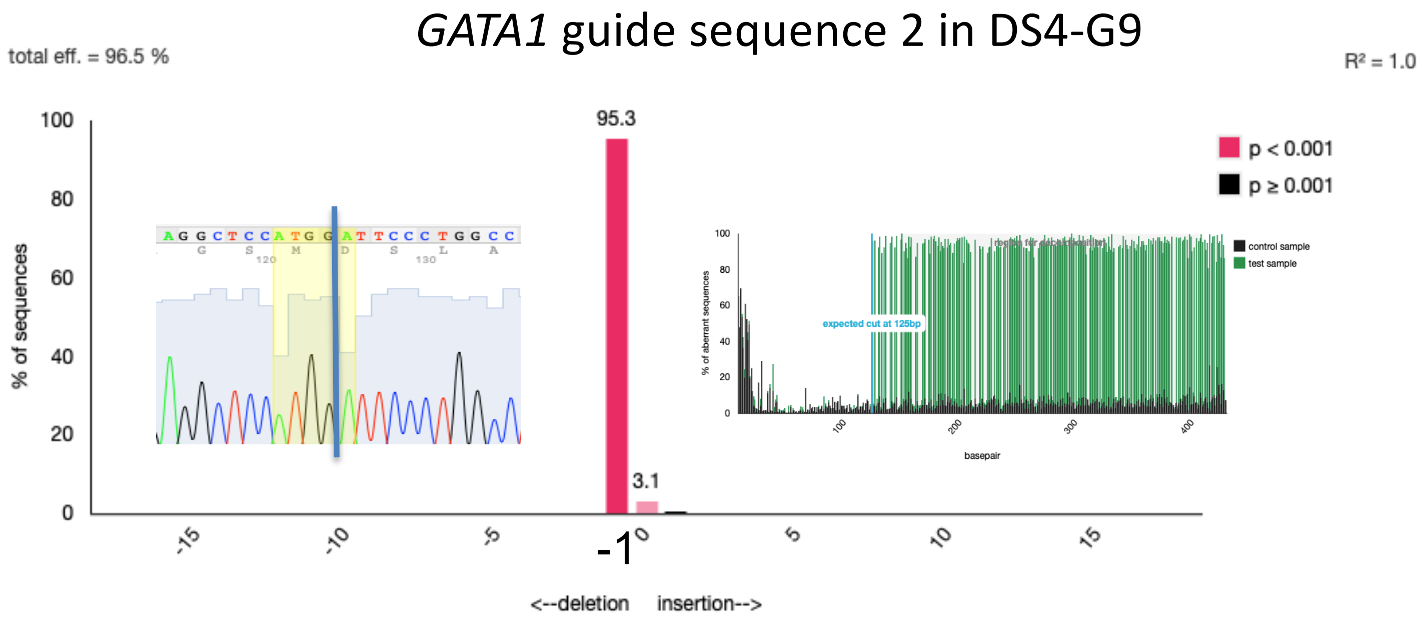


```
CTCGCAGGTTAATCCCCAGAGGCTCCATGGAGTTCCCTGGCCTGGGG Wild-type
CTCGCAGGTTAATCCCCAGAGGCTCCATGGA-TTCCCTGGCCTGGGG Allele 1
```

Supplementary Fig. 2 Analysis of *GATA1* mutation in iPSC line DS4. Plots of TIDE (Tracking of Indels by Decomposition) analysis showing 98.2% allelic mutation frequency of a clone with 2 bp deletion and 96.5% mutation frequency in a clone with 1 bp deletion in trisomic iPSC line DS4. Graph inserts on the right show the rise in sequence aberrations in relation to the CRISPR/Cas9 cut site. Sanger sequencing chromatograms showing the sequence around the cut site (marked with a blue line) are provided. Sequence alignment in the bottom shows the allelic analysis of mutant and wild-type alleles.

Supplementary Fig. 3

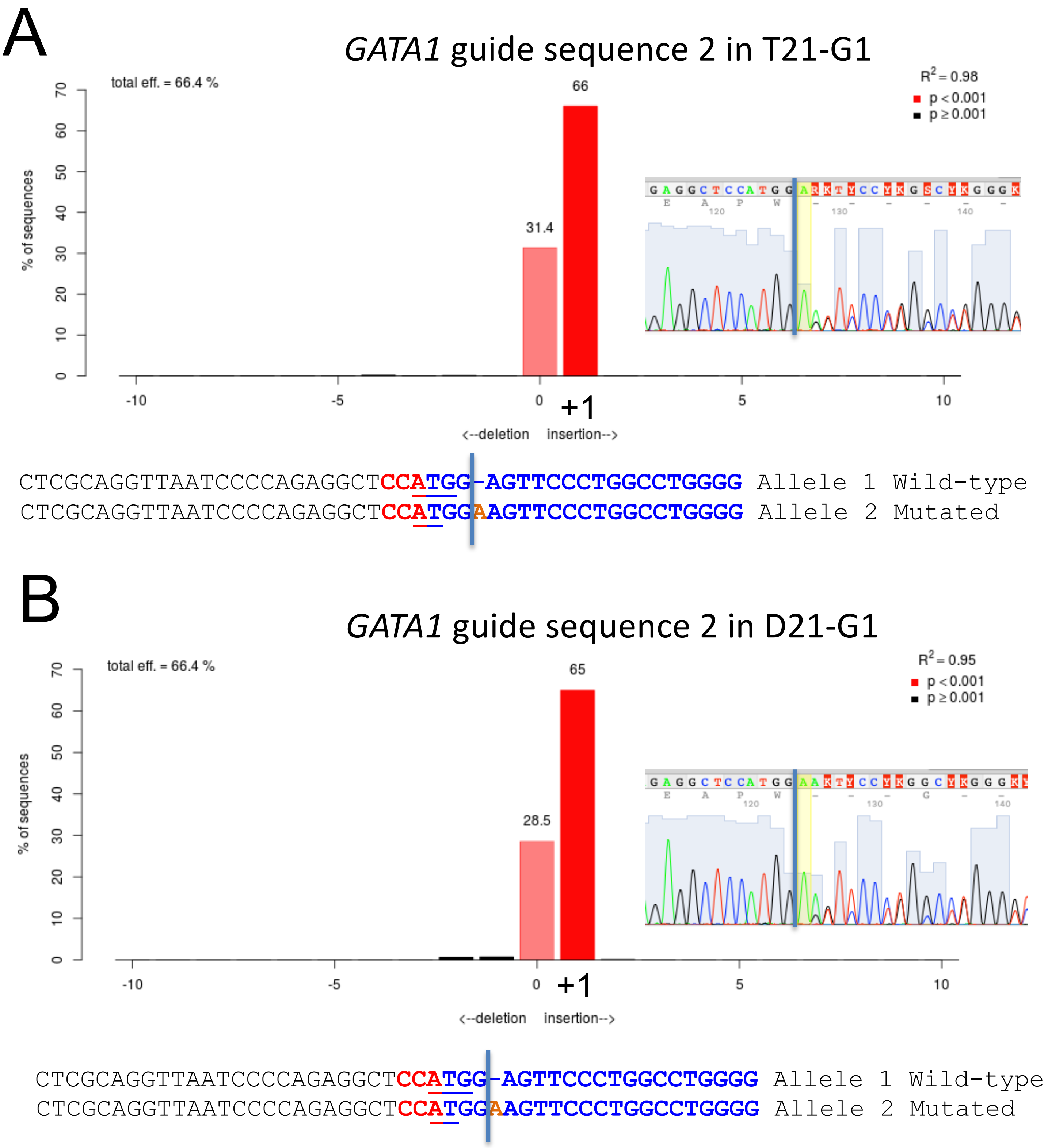

Supplementary Fig. 3 Analysis of *GATA1* mutation in iPSC lines T21 and D21. Plots of TIDE (Tracking of Indels by Decomposition) analysis showing 66.4% allelic mutation frequency in clones with 1 bp insertion in isogenic iPSC lines with trisomy 21 (T21-G1) and disomy 21 (D21-G1). Sanger sequencing chromatograms showing the sequence around the cut site (marked with a blue line) are provided. Sequence alignment in the bottom shows the allelic analysis of mutant and wild-type alleles.

Supplementary Fig. 4

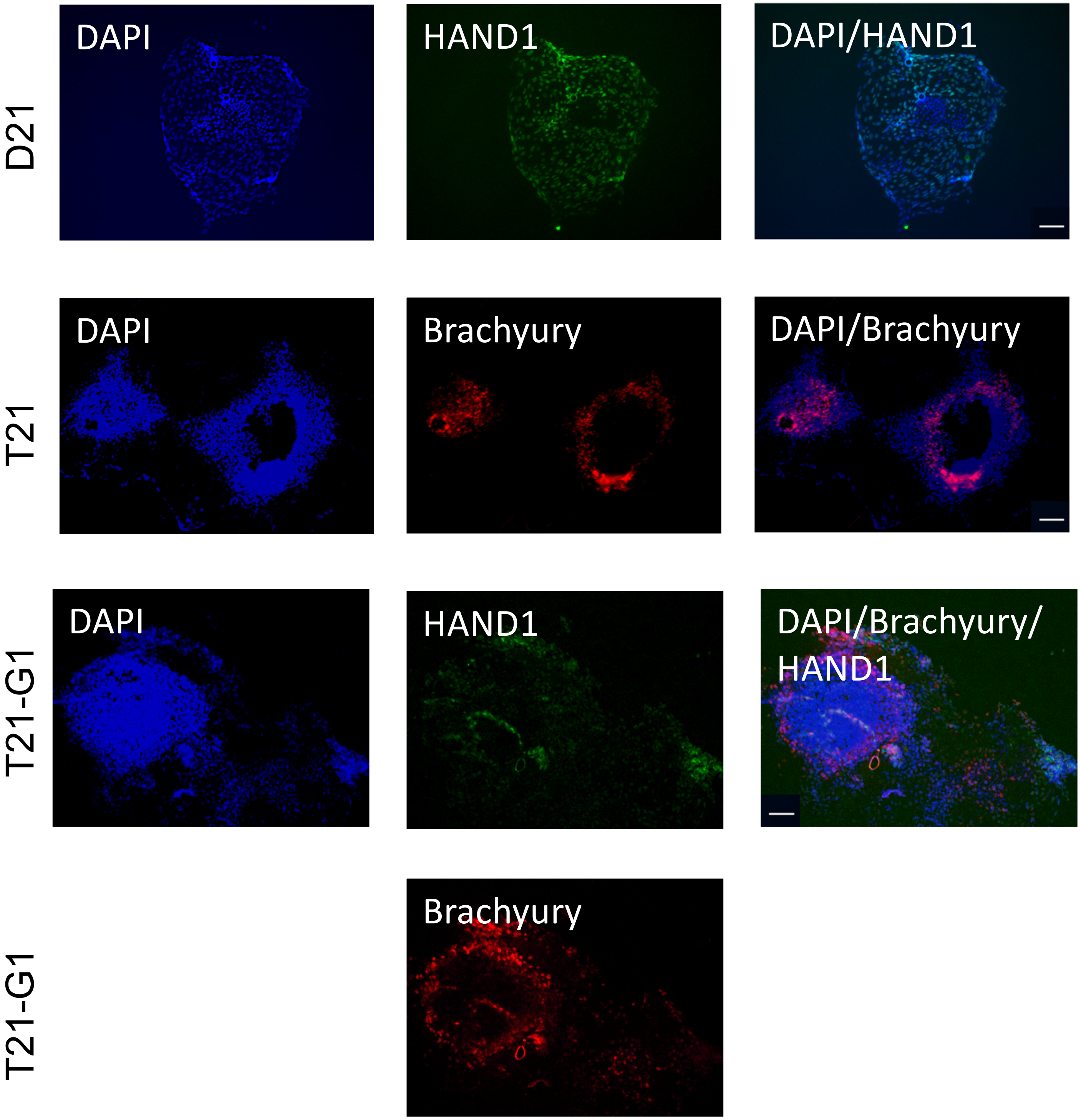

Supplementary Fig. 4 Characterization of mesoderm markers during early stages of iPSC differentiation. Representative immunofluorescence images of indicated iPSC lines stained with mesoderm markers HAND1 (green) and Brachyury (red). Nuclei were stained with DAPI. Scale bar = 100 μm.

Supplementary Fig. 5

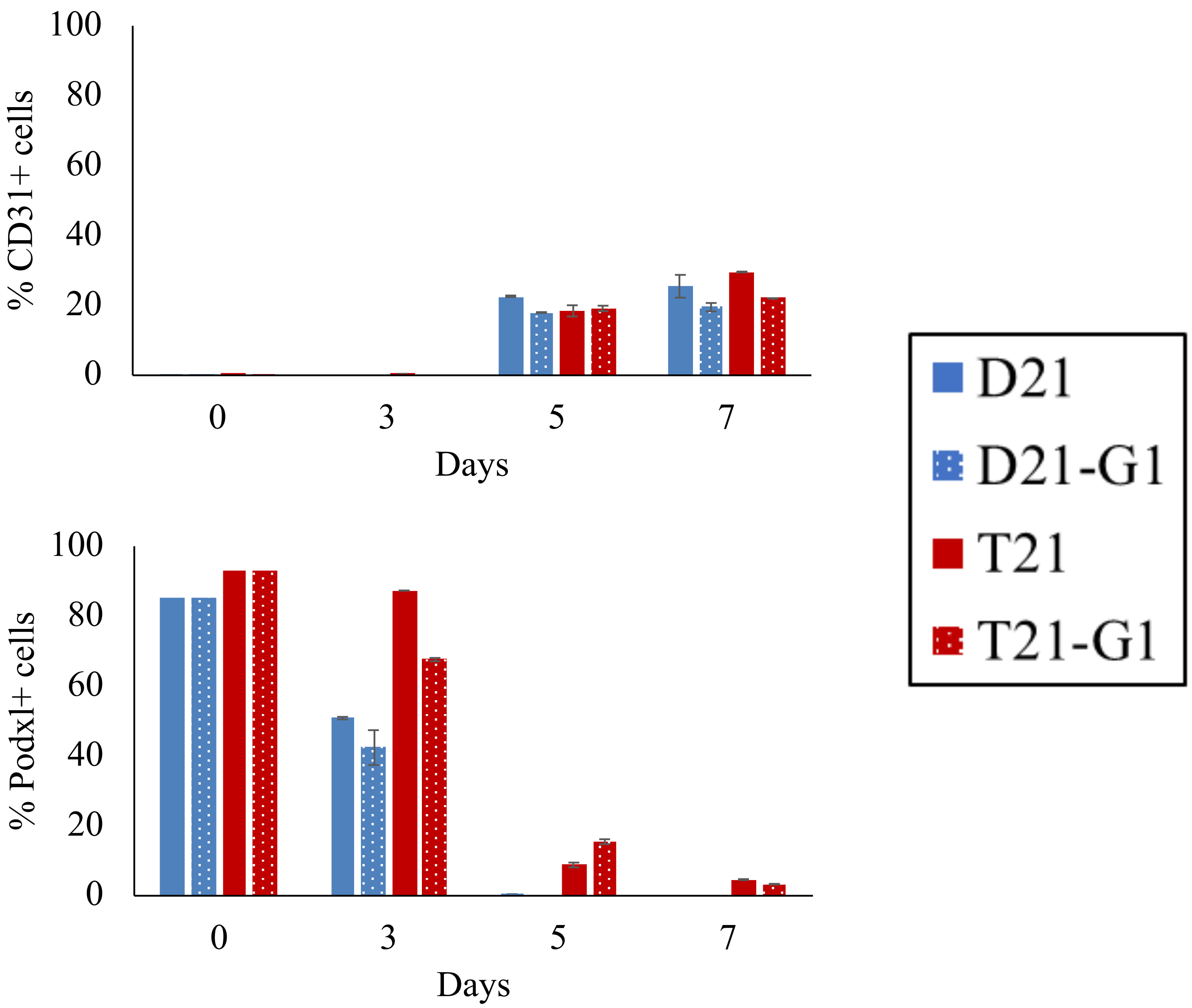

Supplementary Fig. 5 Analysis of hemangioblast markers during hematopoietic differentiation of iPSCs. Graphs showing the percentages of CD31+ and Podxl+ cells at different time points during hematopoietic differentiation of indicated iPSC lines. Error bars denote SD of the mean from two independent experiments.

Supplementary Fig. 6

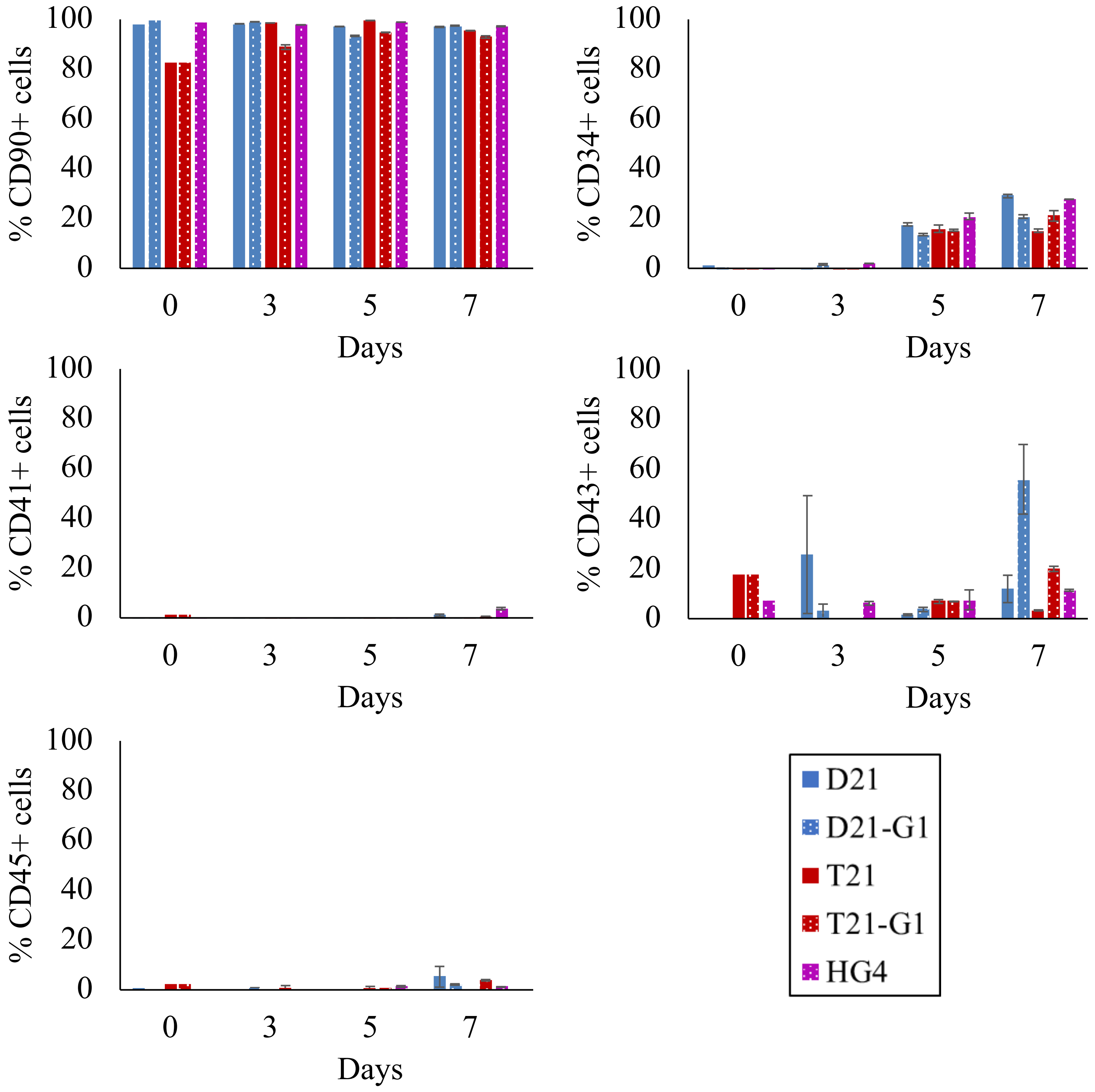

Supplementary Fig. 6 Characterization of hematopoietic markers during iPSC differentiation. Graph showing the percentages of cells positive for distinct hematopoietic markers at different times during hematopoietic differentiation of indicated iPSC lines. Error bars denote SD of the mean from two independ experiments.

Supplementary Fig. 7

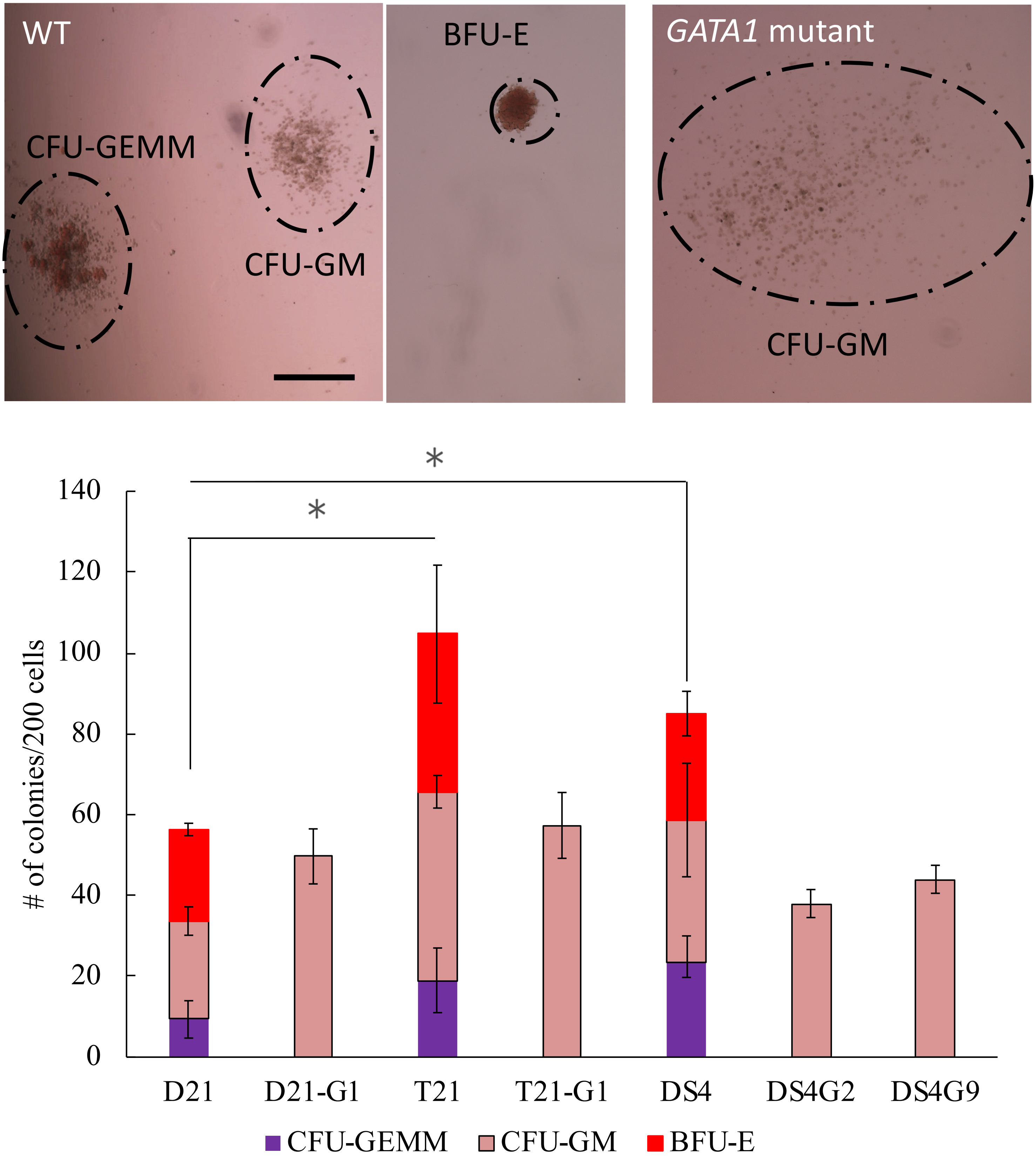

Supplementary Fig. 7 Hematopoietic colony-formation assay. Representative images of colonies developed in methylcellulose colony forming assay from HSPCs. Graph shows the number of colonies of each type identified in indicated iPSC lines, $^*P < 0.05$. Scale bar = 500 µm.

Supplementary Fig. 8

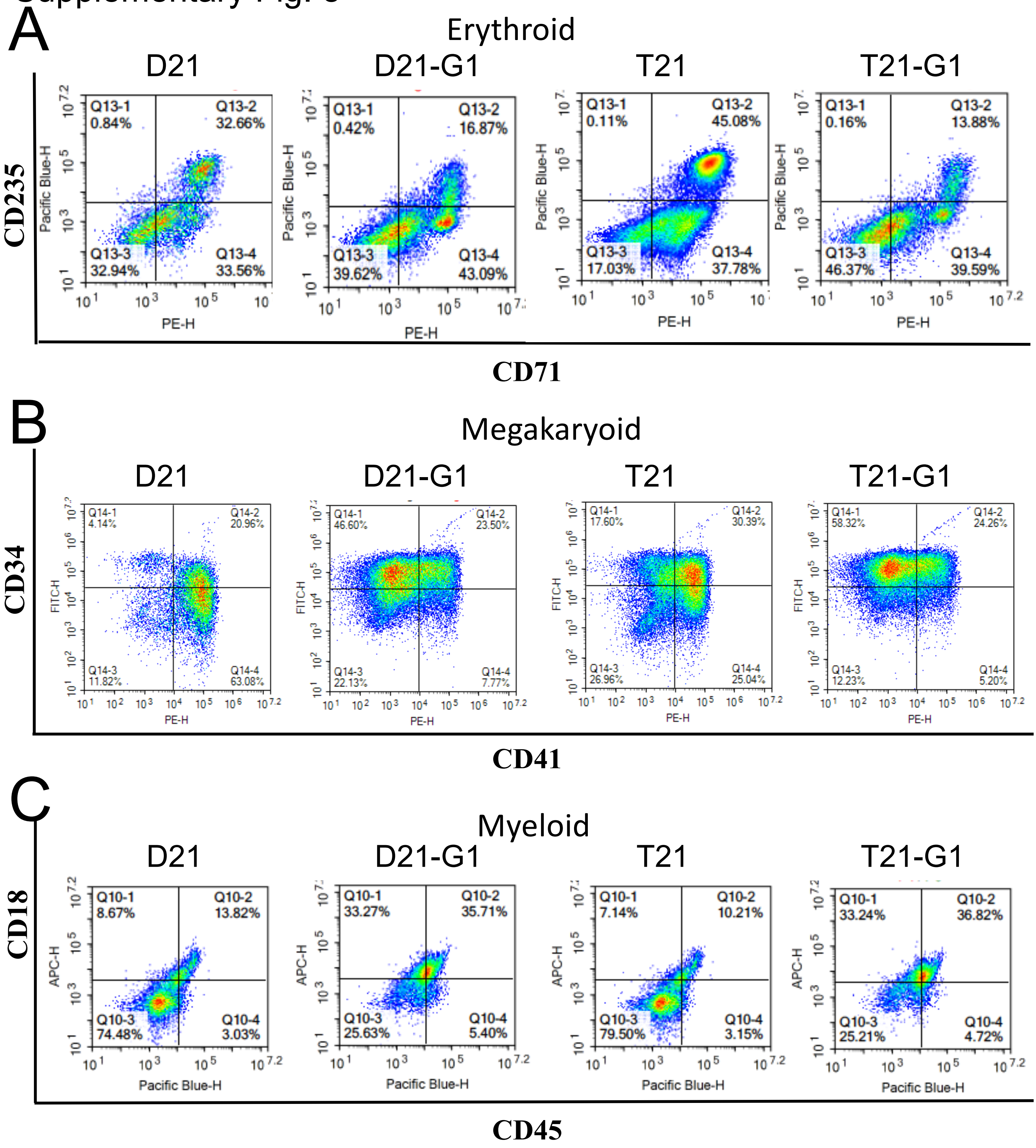

Supplementary Fig. 8 Analysis of erythroid, megakaryoid and myeloid populations in HSPCs generated by hematopoietic differentiation. A) Representative flow plots showing the erythroid population (CD235+CD71+) in HSPCs generated from iPSCs at day 12. **B)** Representative flow plots showing the megakaryoid population (CD34+CD41+) in HSPCs generated from iPSCs at day 12. **C)** Representative flow plots showing the myeloid population (CD18+CD45+) in HSPCs generated from iPSCs at day 12.

Supplementary Fig. 9

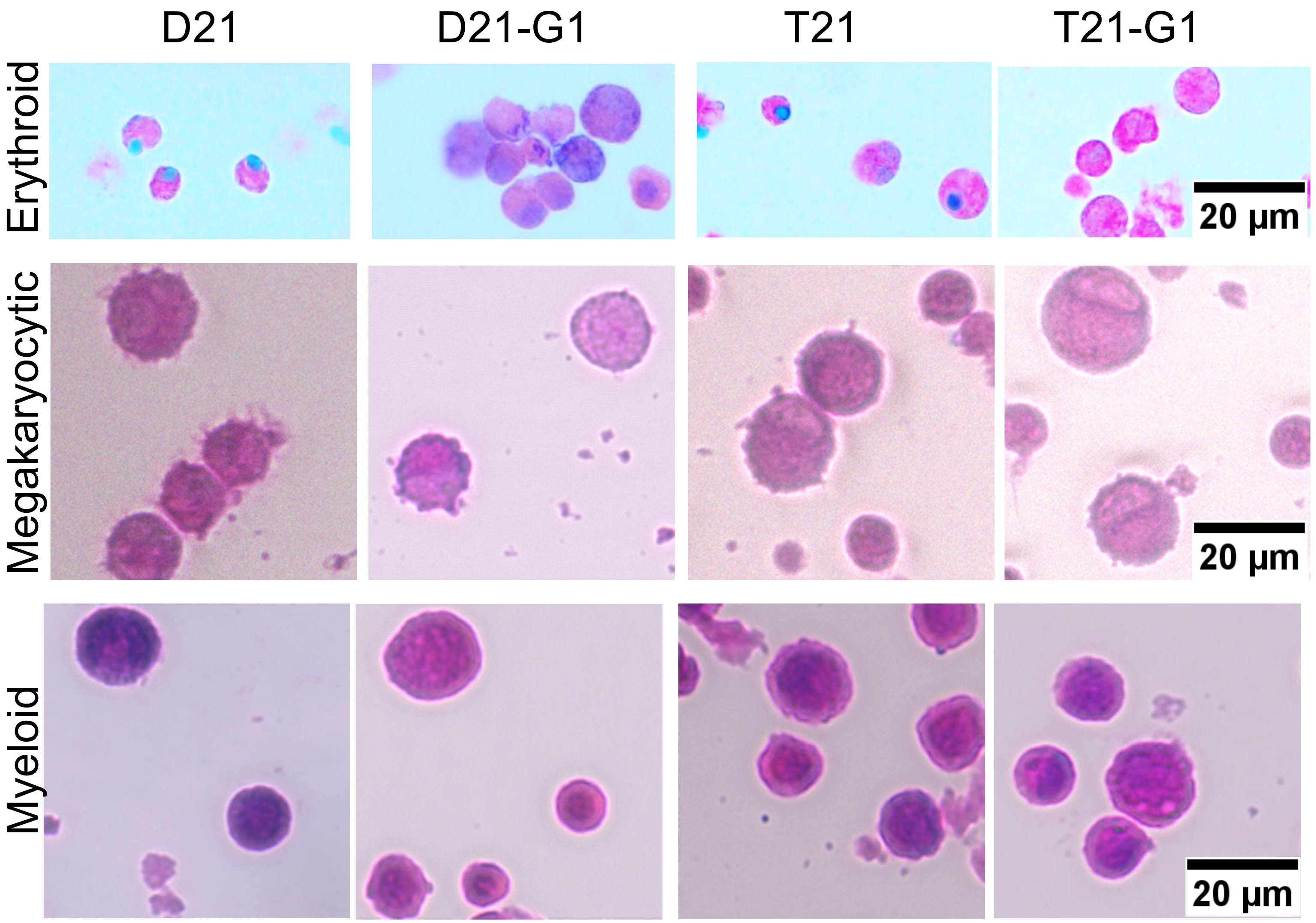

Supplementary Fig. 9 Morphology of erythroid, megakaryocytic and myeloid cells derived by hematopoietic differentiation of iPSCs. Representative images showing the May-Grünwald-Giemsa staining of HSPCs cultured in respective media to promote corresponding lineage expansion. Scale bar = 20 μm.

Supplementary Fig. 10

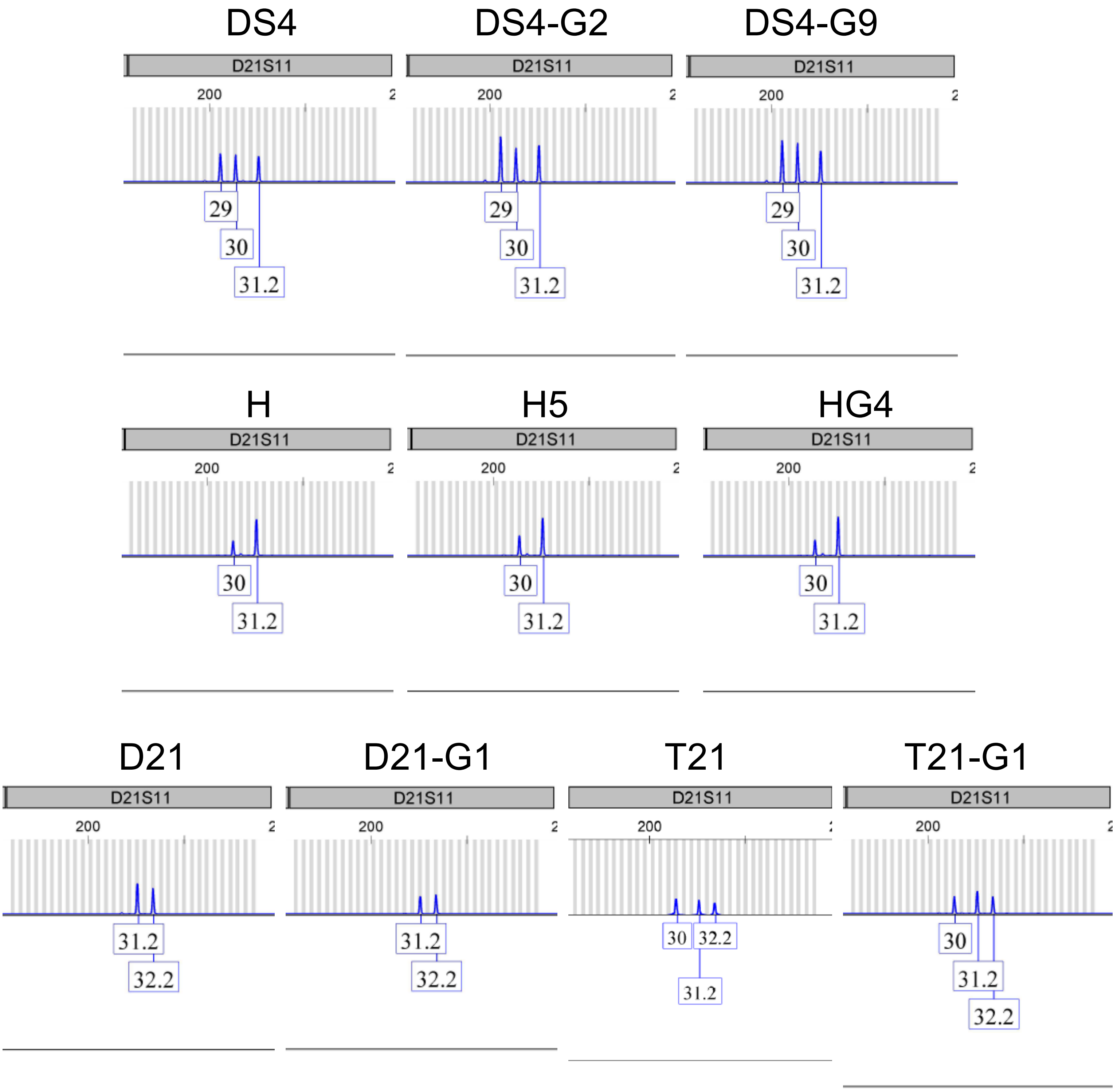

Supplementary Fig. 10 Ploidy analysis of iPSC lines using AmpFLSTR Identifiler PCR Amplification kit. Plots showing the markers at the D21S11 locus on chromosome 21 in the iPSC lines used in the study.

Supplementary Fig. 11

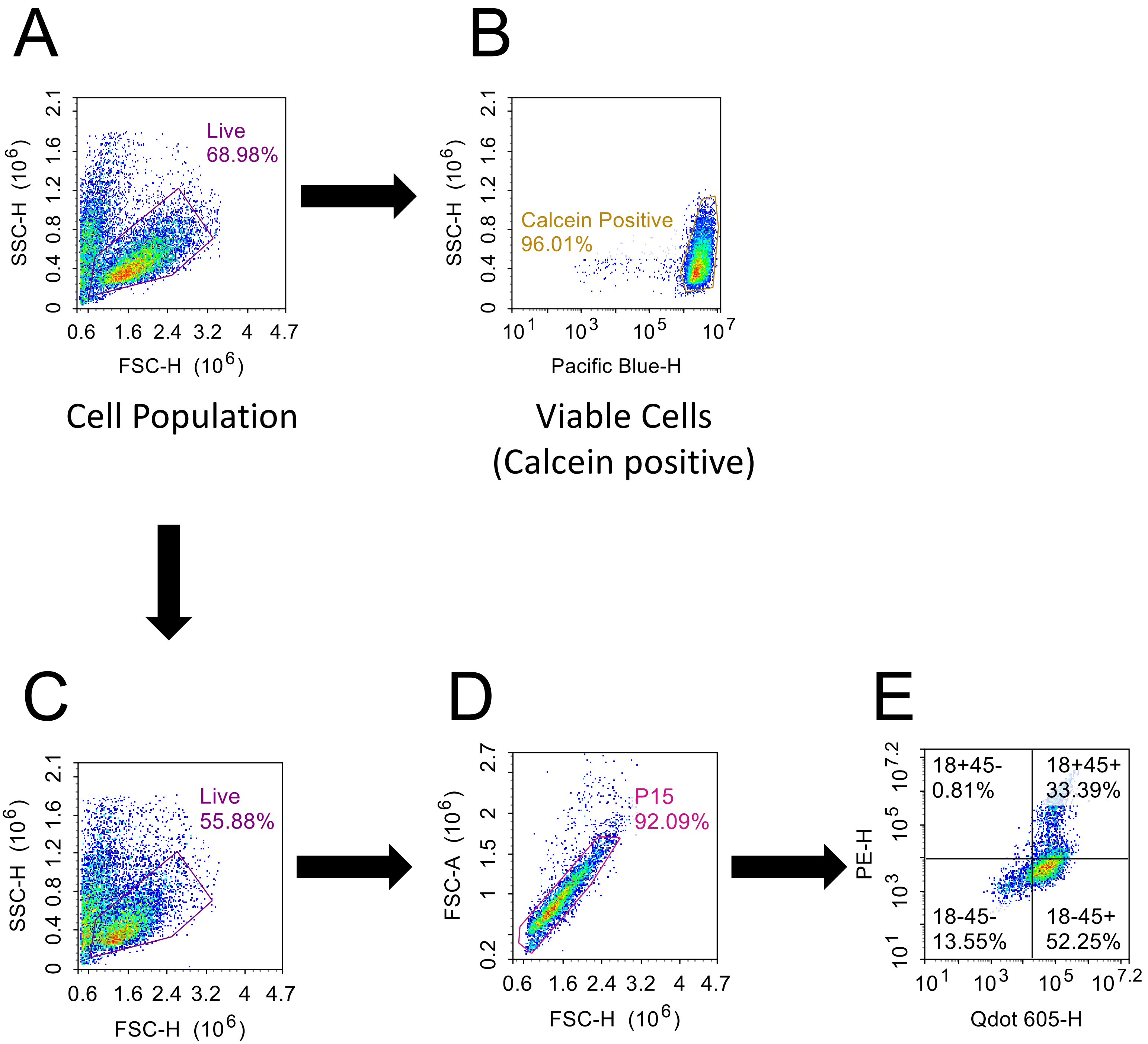

Supplementary Fig. 11 Gating strategy for analysis of live cell population. Representative plots showing the gating strategy used for the analysis of cell populations by flow cytometry.

References

1. Sun, X, Yan, M, Zhang, Y, Zhou, X, Wang, C, Zheng, F*, et al.* (2006). Practical application of fluorescent quantitative PCR on Trisomy 21 in Chinese Han population. *Mol Biol Rep* **33**: 167-173.
